# Differential transcriptome response following infection of porcine ileal enteroids with species A and C rotaviruses

**DOI:** 10.1186/s12985-023-02207-8

**Published:** 2023-10-17

**Authors:** Sergei A. Raev, Molly Raque, Maryssa K. Kick, Linda J. Saif, Anastasia N. Vlasova

**Affiliations:** Center for Food Animal Health Research Program, Department of Veterinary Preventive Medicine, College of Veterinary Medicine, Department of Animal Sciences, College of Food Agricultural and Environmental Sciences, The Ohio State University, Wooster, OH 44677 USA

**Keywords:** Rotavirus C, Glycosyltransferases, Sialic acid, Glycans, Sialyltransferases, Differently expressed genes, Transcriptome analysis, Histo-blood group antigens

## Abstract

**Background:**

Rotavirus C (RVC) is the major causative agent of acute gastroenteritis in suckling piglets, while most RVAs mostly affect weaned animals. Besides, while most RVA strains can be propagated in MA-104 and other continuous cell lines, attempts to isolate and culture RVC strains remain largely unsuccessful. The host factors associated with these unique RVC characteristics remain unknown.

**Methods:**

In this study, we have comparatively evaluated transcriptome responses of porcine ileal enteroids infected with RVC G1P[1] and two RVA strains (G9P[13] and G5P[7]) with a focus on innate immunity and virus-host receptor interactions.

**Results:**

The analysis of differentially expressed genes regulating antiviral immune response indicated that in contrast to RVA, RVC infection resulted in robust upregulation of expression of the genes encoding pattern recognition receptors including RIG1-like receptors and melanoma differentiation-associated gene-5. RVC infection was associated with a prominent upregulation of the most of glycosyltransferase-encoding genes except for the sialyltransferase-encoding genes which were downregulated similar to the effects observed for G9P[13].

**Conclusions:**

Our results provide novel data highlighting the unique aspects of the RVC-associated host cellular signalling and suggest that increased upregulation of the key antiviral factors maybe one of the mechanisms responsible for RVC age-specific characteristics and its inability to replicate in most cell cultures.

**Supplementary Information:**

The online version contains supplementary material available at 10.1186/s12985-023-02207-8.

## Introduction

Rotavirus (RV) infection is the most common cause of severe gastroenteritis and the associated mortality in infants and young children and animals worldwide [1, 2]. Species A RV (RVA) was historically considered to be the most prevalent and pathogenic RV species, associated with > 90% of RV gastroenteritis cases [3]. However, there is growing evidence that species C RV (RVC) is a significant cause of infectious diarrhea in humans and animals [4–7]. While RVAs mostly affect piglets or young children after weaning [8, 9], RVC diarrhea is highly prevalent among nursing, 1–10-day-old piglets, causing significant economic losses to farmers and the pork industry [4, 10, 11]. Recent data suggest that reassortant RVC strains circulate in northeast Asia in both the human and swine populations [12]. In contrast to RVAs, most attempts to isolate or serially propagate RVCs of human or animal origin in continuous or primary cell lines were unsuccessful [13]. The lack of robust cell culture system has limited the ability to study RVC pathogenesis, immunity and to develop or evaluate vaccination/therapeutic approaches. Thus, there is an urgent need in identification of host factors associated with the limited ability of RVC to replicate in vitro.

The main targets for RV infection are mature enterocytes – absorptive intestinal epithelial cells (IECs) located at the tips of intestinal villi [14]. Several molecules on enterocytes and other IECs such as integrins [15], sialic acids (SAs) [16], gangliosides [17], *N*- and *O*-glycans [18–20], heat-shock cognate protein (hsc70) [21] and tight junction proteins [22] are recognized by RV spike protein (VP4) and serve as ligands for RV attachment and entry to IECs [23]. More specifically, histo-blood group antigens (HBGAs) and SA-containing molecules have been demonstrated to serve as attachment factors during RVA and RVC infection [24]. SA-containing molecules play a key role in RV attachment with a genotype-specific manner [24–26]. For example, RVA G9P[13] and RVC Cowden G1P[1] were shown to replicate to higher levels in ileal enteroids after terminal SA removal by neuraminidase treatment [24, 25] suggesting that SAs may mask other attachment sites from RV binding. However, it remains unknown whether replication of these viruses leads to modulation of SA biosynthesis and metabolism.

Intestinal epithelium is protected from the luminal content by the mucus layer [27]. Mucus major organic components—mucins [28]—have been demonstrated to provide protection against RV infection in vivo and in vitro [29, 30] functioning as both the physical barrier and an innate immune factor. Despite its significance, little is known about the host mucin-related transcriptome response in cells infected with RVA or RVC. For example, increased production of MUC2 during an RVA infection has also been found to be one of defence mechanisms in germ-free (GF) mice [29], while changes in glycosylation profiles of the intestinal mucins were observed in the course of RVA infection of mice [31].

The aim of this study was to comparatively evaluate the effects of RVA and RVC infection of porcine ileal enteroids on the expression of genes associated with innate immune response and metabolism of mucins, glycans and gangliosides.

## Materials and methods

### Rotavirus A and C infection

Porcine ileal enteroids (PIEs) were maintained as described previously [25]. For RV infection sterile gnotobiotic pig intestinal contents containing RVA RV0084 G9P[13] [4], RVA OSU G5P[7] and RVC Cowden G1P[1] [32] were used in the study as previously described [24]. Briefly, Intestinal contents were diluted at a 1:10 ratio in sterile Minimal Essential Media (MEM Gibco; Life Technologies, Grand Island, NY, United States). Contents were then centrifuged at 2,095 × g for 10 min at 4 °C and filtered through a 0.2 mm filter. All RV strains were preactivated with 10 ug/ml of trypsin derived from porcine pancreas(Thermo Fisher Scientific, USA) for 30 min at 37 °C. Virus titers were adjusted to desired multiplicity of infection (MOI) 1.0. Infected PIEs were harvested and frozen at 0 and 24 h post infection. The harvested PIEs were kept at − 80 °C until RNA was extracted from homogenized cells.

*RNA sequencing.* RNA samples were extracted from PIEs infected with RVA (OSU and RV0084) and RVC (Cowden) strains and non-infected mock-treated controls using MagMAX™ Viral/Pathogen Nucleic Acid Isolation Kit (Thermo Fisher Scientific) following the manufacturer’s instruction. For RNA quantification we used Nanodrop spectrophotometer (Promega, USA) RNA. All samples had concentrations of > 36.00 ng/ul and RNA integrity number (RIN) values above 7 [33]. The isolated RNA samples were stored at -80 degree. RNA samples were submitted to Psomagen (USA) for *Sus scrofa* whole transcriptome sequencing and identification of Differentially Expressed Genes (DEG). Briefly, purified RNA samples were randomly fragmented and reverse transcribed [34].

### Expression profile comparison

The quality of the raw sequences was evaluated by FastQC v0.11.7. The read count value of known genes obtained through -e option of the StringTie were used as the original raw data [35]. Raw read count data were transformed to FPKM (Fragment per Kilobase of transcript per Million mapped reads). In order to remove adapter sequences and bases with low base quality reads were trimmed [36] (by using Trimmomatic 0.38) and obtained fragments with 200–400 bp size were mapped (by using HISAT2 v. 2.1.0) to the reference genome [37]. Statistical analysis was performed using fold change (fc), exactTest using edgeR [38]. Expression profile was calculated for each sample and transcript/gene as read count and FPKM. Expression profiles for DEG were normalized based on length and depth of coverage by using the FPKM normalization value. The significant results were selected on conditions of exactTest raw *p*-value < 0.05.

### Ingenuity pathway analysis (IPA)

The web-based pathway analysis tool, IPA (Qiagen, Germany) was used to identify biological functions and molecular networks modulated in PIEs infected with RVA and RVC. For IPA only significantly affected genes (fold change cut off ± 2 ≥ or ≤ 2) were uploaded. The Ingenuity Pathways Knowledge Base (IPKB) was used to identify relevant networks and canonical pathways. The significance of the association between the genes from the data set and the canonical pathways was measured as described in IPA. Differently expressed genes were mapped to biological functions in order to build the functional analysis. Fischer’s exact test was used to calculate a *p* value for each biological function assigned to that network. The activation Z-score makes predictions about potential regulators by using information about the direction of gene regulation [39].

## Results

### RVC caused an extensive alteration of PIE gene expression with a robust upregulation of innate immune signaling pathways

**Fig. 1:**
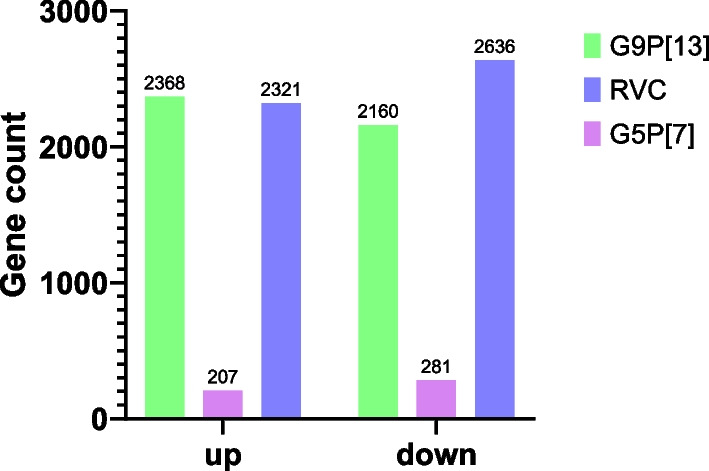
Summary of the total gene numbers that were either upregulated or downregulated (DEGs)(|fc|≥ 2 and raw *p* < 0.05). Non-infected PIEs were used as negative controls for all viruses. Total number of genes is placed at the top of the individual bars and split according to representation of upregulated and downregulated genes

PIE infection with G5P[7], G9P[13] and RVC resulted in drastically variable total numbers of DEGs (Fig. 1). While RVC and G9P[13] modulated higher numbers of genes (4,957 and 4,528, respectively), G5P[7] infection led to significant modulation of only 488 genes..

**Fig. 2:**
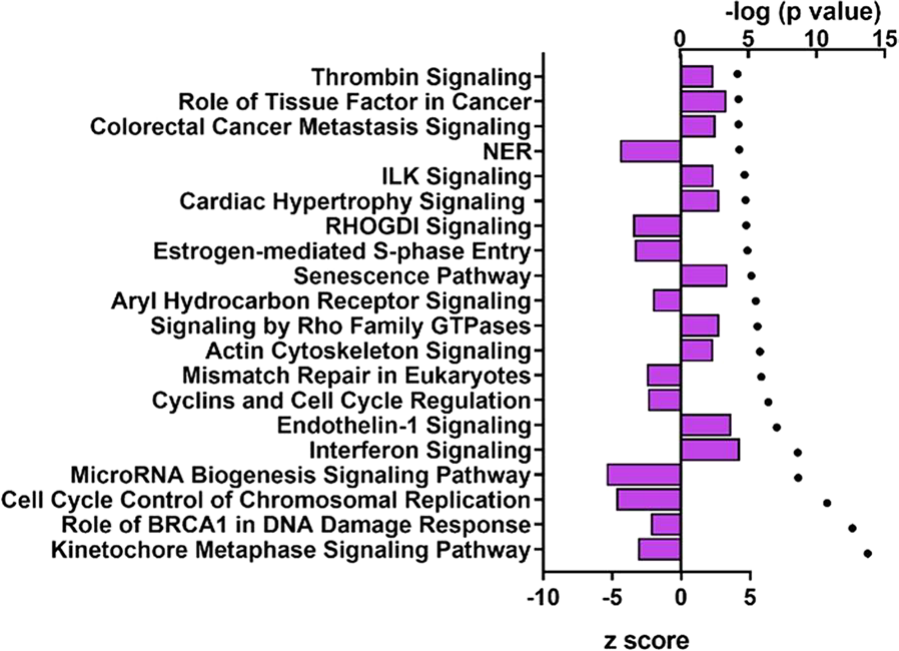
Top20 canonical pathways modulated in PIEs following the RVC infection vs control (non-infected PIEs) (performed using Qiagen IPA). Canonical pathways listed are as followed: Thrombin signaling, role of tissue factor in cancer, colorectal cancer metastasis signaling, nucleotide excision repair (NER), integrin-linked kinase (ILK) Signaling, cardiac hypertrophy signaling, RHOGDI signaling, estrogen-mediated S-phase entry, senescence pathway, aryl hydrocarbon receptor signaling, signaling by Rho family GTPases, actin cytoskeleton signaling, mismatch repair in eukaryotes, cyclins and cell cycle regulation, endothelin-1 signaling, interferon signaling, microRNA biogenesis signaling, cell cycle control of chromosomal replication, role of BRCA1 in DNA damage response, kinetochore metaphase signaling. Canonical pathway analysis was conducted using the IPA library and shows the most significant contributions through the input data set. For canonical pathway analysis the—log (*p*-value) > 1.3 was taken as threshold, the z-score > 2 was defined as the threshold of significant activation, whilst z-score <  − 2 was defined as the threshold of significant inhibition. Bars indicate the activation z-score; blue symbols represent *p* values in log10 format

As shown in Fig. 2, infection of PIEs with RVC was mostly associated with significant modulation of canonical pathways related to cellular growth, cancer development and interferon response. The most prominent downregulation was observed for the genes from the miRNA biogenesis signalling pathway which coincided with downregulated expression of genes of cell cycle control of chromosomal replication, estrogen-mediated S-phase entry, cyclins and cell cycle regulation and kinetochore metaphase signalling pathways indicating an overall predicted downregulation of cellular proliferation. In contrast, interferon signaling pathway was upregulated.

**Fig. 3:**
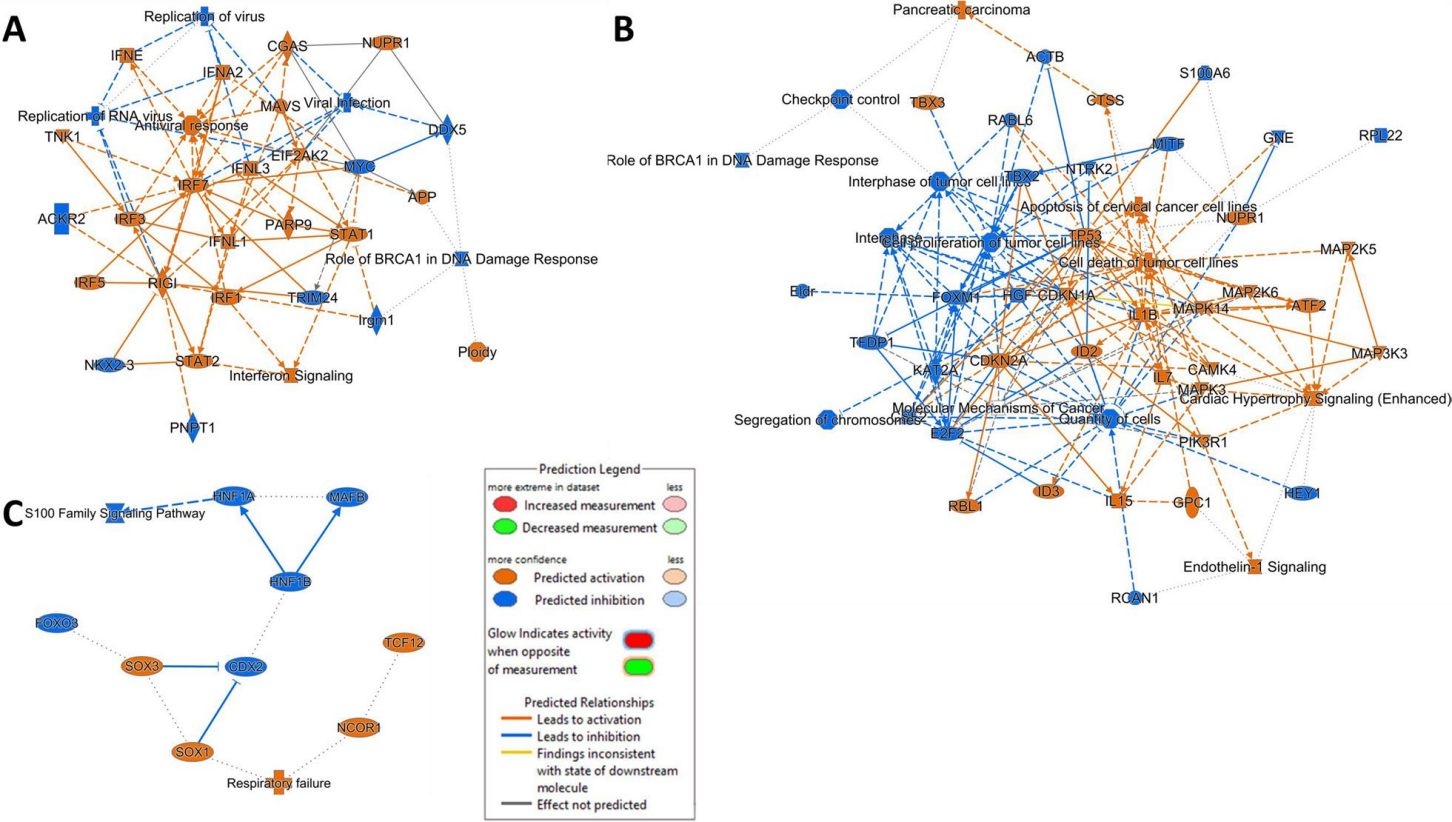
Graphical summary of PIE response following RVC (**A**), G9P[13] (**B**), and G5P[7] (**C**) infection vs control (non-infected PIEs) depicts the associations between the mostly modulated biological themes, creating a coherent and comprehensible synopsis of the analysis. The graphical summary includes entities such as canonical pathways, upstream regulators, diseases and biological functions. **A** The graphical summary for RVC infection revealed two affected canonical pathways (Role of BRCA1 in DNA damage response; and Interferon signalling – Top 5 canonical pathways in Fig. 2) with regulators of the immune response including STAT1, STAT2, DDX5. **B** The data analysis of the PIE transcriptome response following RVA G9P[13] infection indicated a prominent association of the top modulated canonical pathways (Endothelin signalling, Role of BRCA1 in DNA damage with a variety cancer/cell proliferation-associated genes). **C**. In contrast, only a few genes were connected with S100 family signalling pathway in PIEs infected with RVA G5P[7]

**Fig. 4:**
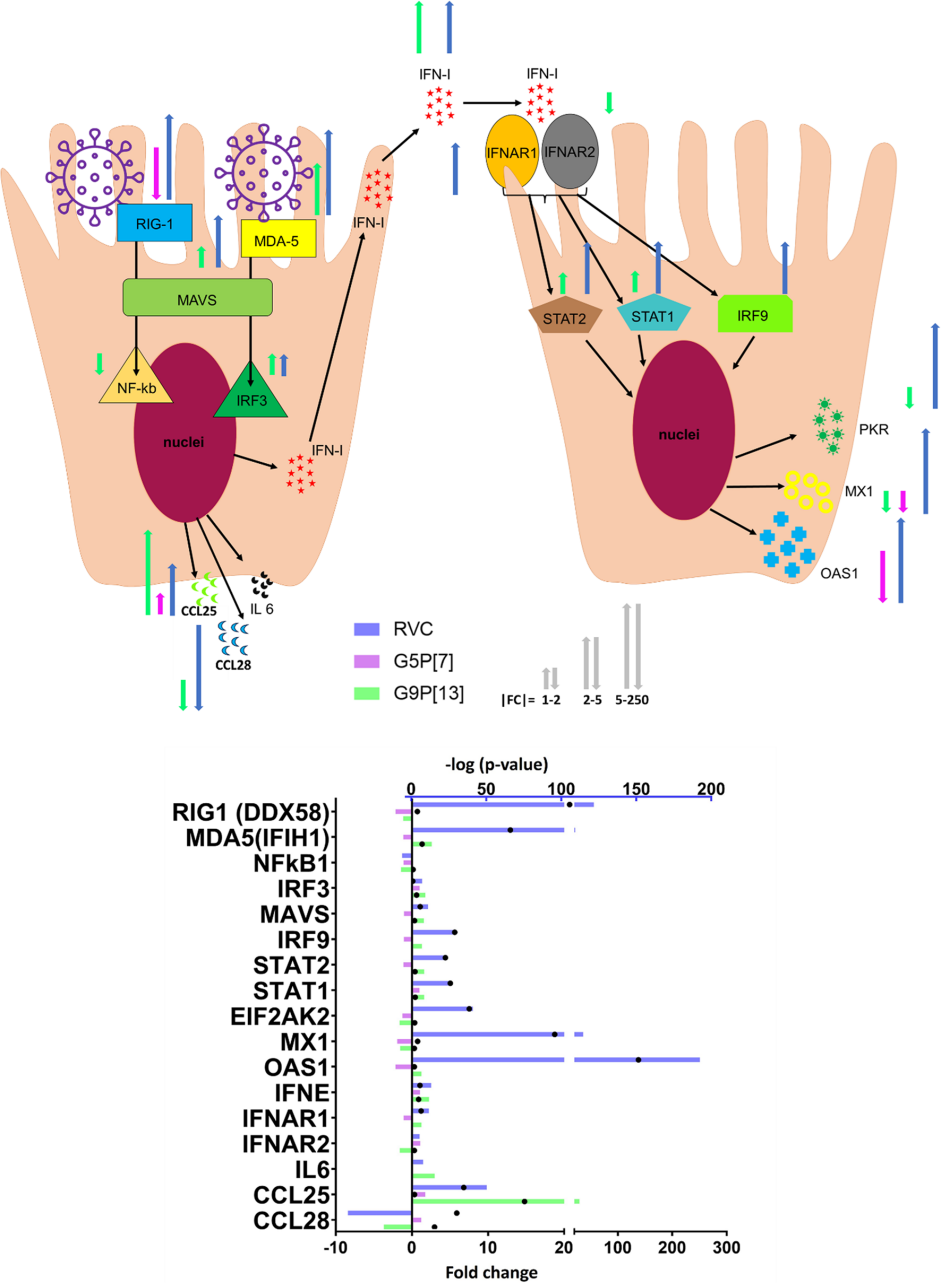
Change of expression of genes associated with immune response in infected PIEs. Bars indicate fold change (FC) range difference between infected PIEs vs uninfected control. Black circles represent the significance (*p*-value < 0.05, i.e. 1.3 in − log10 format). Arrows indicate the significant fold change range

Further, IPA analysis was used to identify the associations between the most modulated canonical pathways, upstream regulators, diseases and biological functions indicating that infection of PIEs with RVC, but not RVA, was associated with a robust predicted activation of antiviral response (Figs. 2, 3, 4, Additional File 1). More specifically, this analysis highlighted a predicted activation of interferon signaling (interferon regulatory factors 1, 3, 5 and 7), mitochondrial antiviral signaling (MAVS) (Fig. 3a). MAVS is an adaptor protein that coordinates the activation of IFN inducing pathways, interferon production (IFN-epsilon, alpha 2, lambda 3) and their interactions with retinoic acid-inducible gene I (RIG-I) receptors known to be responsible for mediating the transcriptional induction of type I interferons [40]. In addition, our study revealed that following RVC infection RIG-1 and interferons were predicted to activate molecules that activate the innate antiviral immune response: signal transducer and activator of transcription type 2 (STAT2) and STAT1 (Fig. 3a) [41, 42]. Our analysis suggested that predicted inhibition of “viral infection”, “replication of virus” and “replication of RNA virus” could be associated with the aforementioned components of the innate immunity (RIG-1, STAT2, interferons) and DEAD-box protein 58 (DDX58) (Fig. 4)—a multifunctional protein responsible for recognition of double stranded RNA [43]. In contrast, graphical summaries for PIEs infected with the two RVA strains (Figs. 3B, C and 4) did not reveal a strong activation of virus replication/immune response. Analysis of the profiles of DEGs associated with immune response indicated a significant downregulation of a gene encoding RIG1 (DDX58) (Fig. 4) a molecule responsible for recognizing virus-infected cells after G5P[7] infection while the infection with G9P[13] led to only marginal downregulation. In contrast, the expression of both RIG1 and -MDA5 (another principal sensor of viral dsRNA) -encoding genes was significantly upregulated after RVC infection. This difference was associated with more robust expression of transcription factor IRF3 and NF-kB-encoding genes, MAVS as well as IFN type 1 and 3-encoding genes expression which, in turn, was associated with a significant upregulation of IRF9, STAT1 and STAT2—encoding genes in RVC and G9P[13] infected PIEs. These effects also coincided with a robust upregulation of OAS-1, MX-1 and (to a lesser extent) EIF2AK2 (PKR)—genes encoding essential proteins involved in the innate immune response to viral infection [44, 45]. In contrast, infection with RVA strains G9P{13] led to significant downregulation of the expression of *MX1* (both viruses) and EIF2AK2 (PKR) (G9P[13] only).

### RVA and RVC infection led to a significant upregulation of the genes encoding transmembrane and secreted mucins

**Fig. 5:**
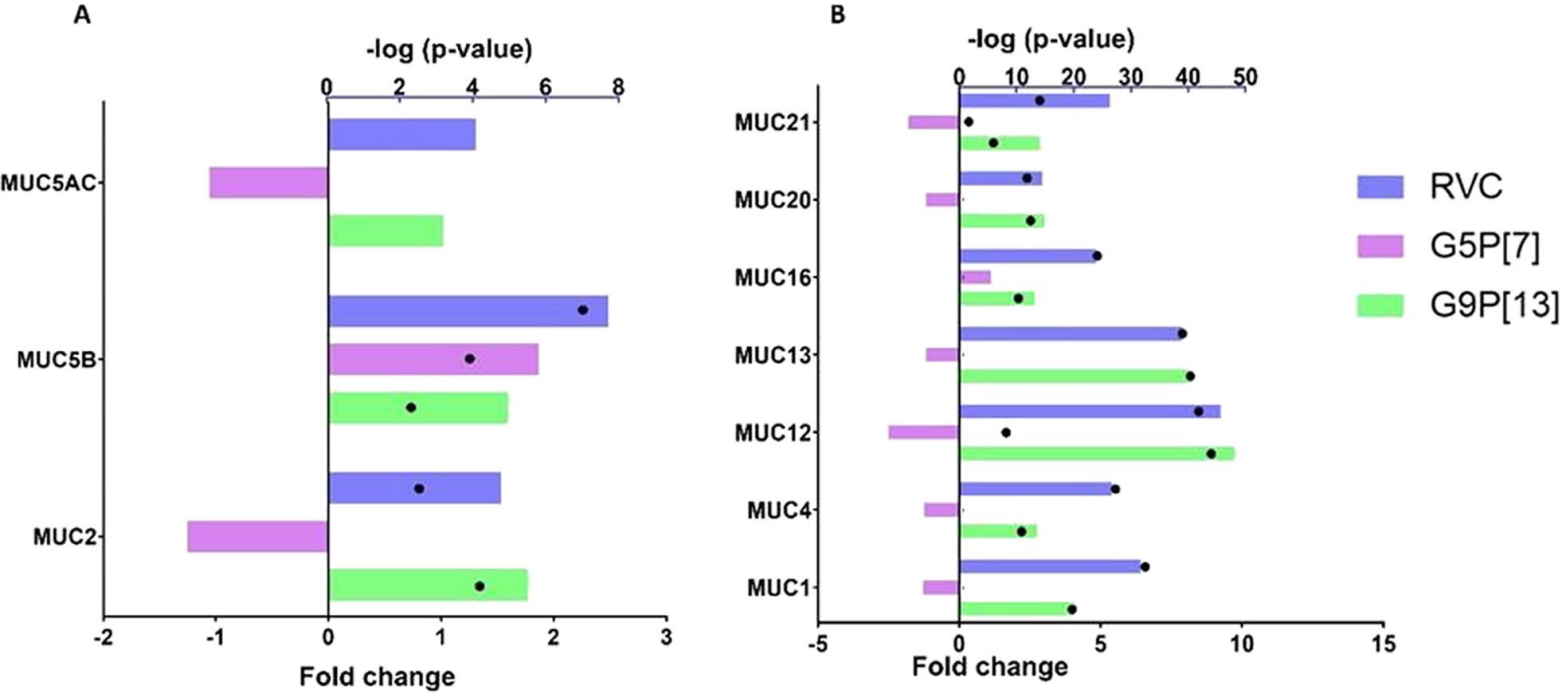
Change of expression of genes encoding secreted gel-forming (**A**) and transmembrane (**B**) mucins in PIEs infected with RVC, G9P[13] and G5P[7] versus control (non-infected PIEs). Bars indicate fold change difference between infected PIEs vs uninfected control. Black circles represent the significance (*p*-value < 0.05, i.e. 1.3 in − log10 format)

PIE infection with RVA and RVC led to significant alterations in expression of mucin-encoding genes including secreted gel-forming mucins (Fig. 5, Additional file 1). Expression of the major gel-forming mucin—MUC2 and MUC5AC was significantly upregulated by G9P[13] and RVC whereas infection with G5P[7] led to a marginal downregulation of these genes’ expression. Expression of another gel-forming mucin, MUC5B, was significantly upregulated by all RVs used in this study. Interestingly, we observed a significant upregulation of expression of all transmembrane-mucin encoding genes following infection with G9P[13] and RVC. In contrast, G5P[7] infection decreased expression of transmembrane mucin genes significantly (MUC12, MUC16 and MUC21) or numerically (MUC1, MUC4, MUC13 and MUC20).

### RVA and RVC infection led to a significant modulation of the glycosyltransferase-encoding genes involved in *O*-, *N*-glycan and ceramide biosynthesis

**Fig. 6:**
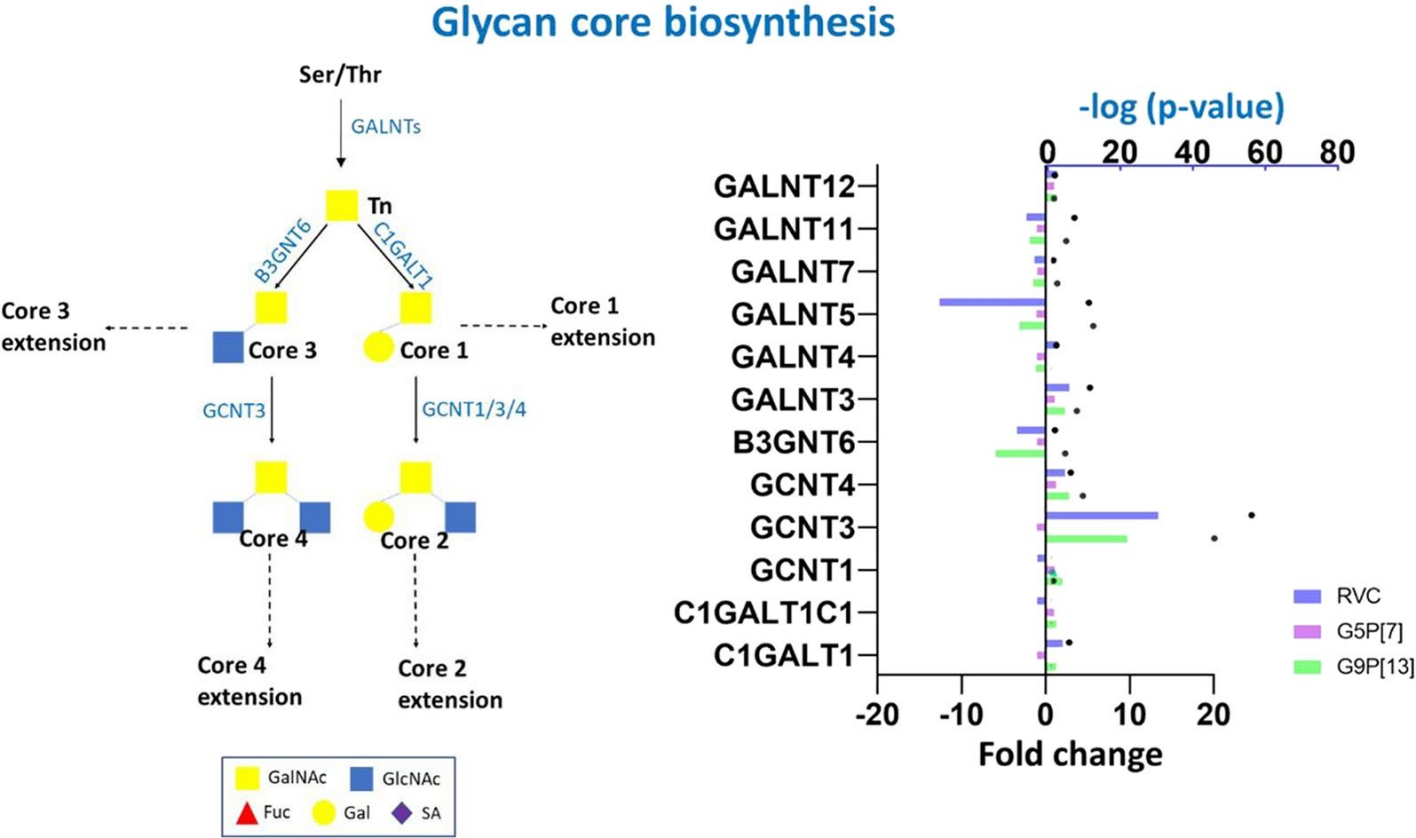
Change of expression of genes encoding glycosyltransferases catalyzing the synthesis of glycan cores [1–4] in PIEs infected with RVC, G9P[13] and G5P[7] versus control (non-infected PIEs). *O*-glycan development is a sequential process which requires the activity of several enzymes belonging to glycosyltransferase family. The initial step of mucin-type *O*-glycosylation in mammals (addition of GalNAc to PTS domain—Tn antigen) is provided by the activity of *N*-acetylgalactosaminyltransferases (ppGalNAc-Ts)—enzymes which are encoded by one of 20 genes. This antigen is further extended to glycan cores by addition of different carbohydrate residues including *N*-acetylgalactosamine (GalNAc), galactose (Gal), *N*-acetylglucosamine (GlcNAc), fucose (Fuc) and SA provided by activity of different glycosyltransferases. These extended glycan core structures can be included in the structure of secreted or transmembrane mucins MUC1, MUC2, MUC5AC, MUC5B, MUC6-8, MUC 11-13 and MUC 16 (79). Bars indicate fold change difference between infected PIEs vs uninfected control. Black circles represent the significance (*p*-value < 0.05, i.e. 1.3 in − log10 format)

Our data indicated (Fig. 6, Additional File 1) a robust modulation of expression of glycosyltransferase-encoding genes. Infection with both, RVC and G9P[13] significantly modulated the expression of genes encoding a family of polypeptide *N*-acetylgalactosamine (GalNAc) glycosyltransferases responsible for the biosynthesis of Tn antigen (GALNT family)—initiation of mucins glycosylation—addition of GalNAc to serine or threonine. In humans, there are 20 known members of this enzyme family. In this study, we identified 9 DEGs (Galnt 1–5, 7, 10–12) following RVA and RVC infection. Among them, expression of six [3–5, 7, 11, 12] genes was significantly modulated. The most prominent effect (downregulation) on the expression of these enzymes was observed for GALNT5 in PIEs infected with RVC and G9P[13]. Similarly, *GALNT11* and *GALNT7* were downregulated but to a lesser extent. However, expression of GALNT3, GALNT4 and GALNT12-encoding genes was significantly upregulated in G9P[13] and RVC-infected PIEs.

Further extension of Tn antigen towards glycan core 1 biosynthesis (provided by C1GALT1 activity) (Fig. 6) was predicted to be upregulated after infection with RVC but not RVA strains. Expression of the genes responsible for glycan core 2 biosynthesis was upregulated in PIEs infected with G9P[13] and RVC, but not G5P[7]. The most prominent modulation of expression of glycosyltransferase-encoding genes associated with glycan-core formation was observed for GCNT3 (plays a key role in both glycan core 2 and 4 biosynthesis) in association with RVC and G9P[13] infection. However, since the expression of B3GNT6-encoding gene was significantly downregulated in PIEs infected with G9P[13] and RVC, we have concluded that infection with these viruses led to predicted activation of glycan cores 1 and 2 biosynthesis. In addition, despite the overall similarity in the modulation of core 2 synthases gene expression between RVC and G9P[13], only the latter significantly upregulated the expression of *GCNT1*. In contrast, the expression of none of glycosyltransferase-encoding genes responsible for glycan core biosynthesis was significantly changed by G5P[7] infection.

**Fig. 7:**
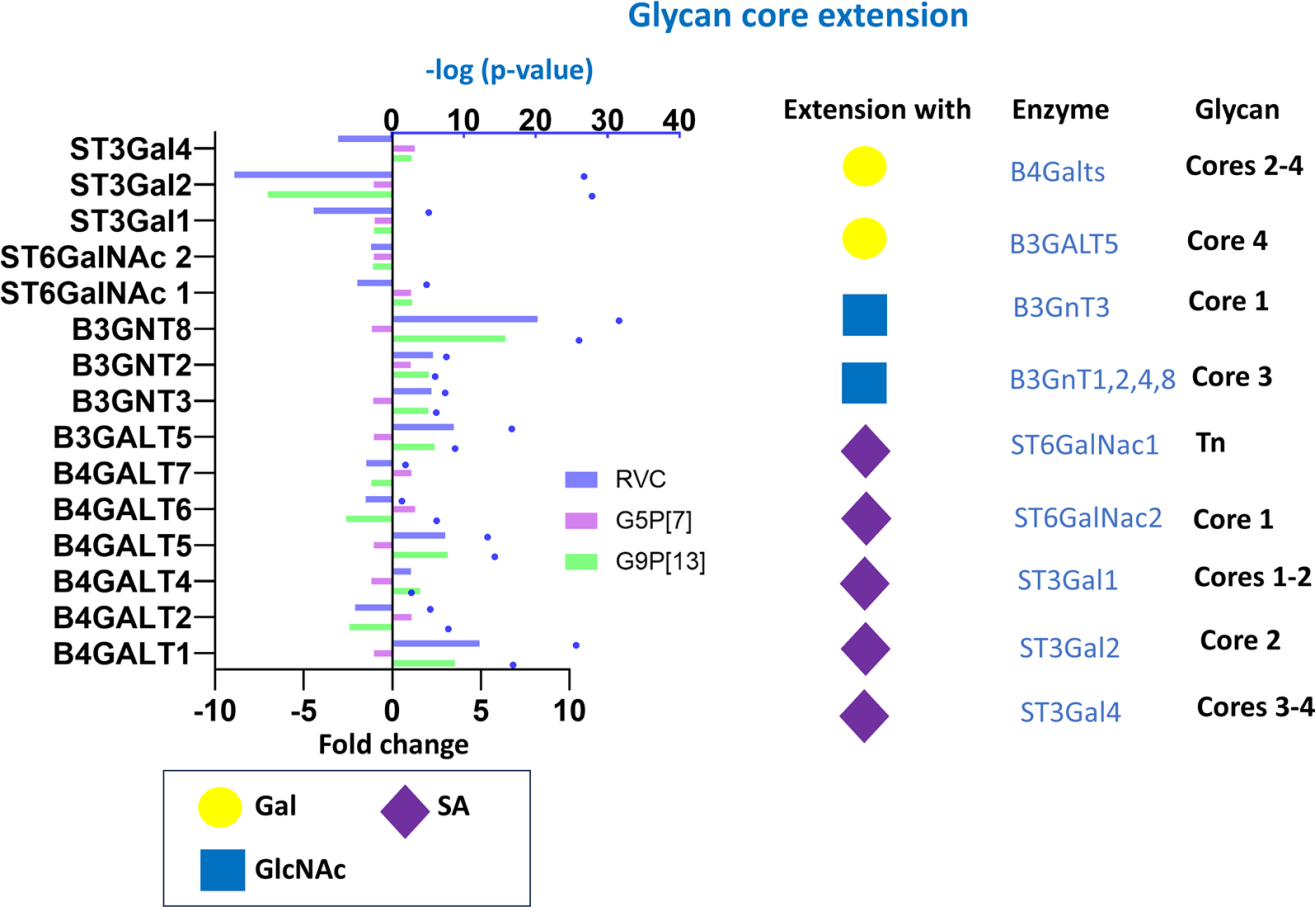
Change of expression of genes encoding glycosyltransferases catalyzing transfer of sugar residues to glycan cores (glycan core extension) in PIEs infected with RVC, G9P[13] and G5P[7] vs control (non-infected PIEs). The peripheral terminal region of glycan cores may include d-galactose (Gal), *N*-acetylglucosamine (GlcNAc) and SA residues whose transfer is provided by several glycosyltransferases in a core-specific manner. Bars indicate fold change difference between infected PIEs vs uninfected control. Blue circles represent the significance (*p*-value < 0.05, i.e. 1.3 in -log10 format)

A similar observation was demonstrated for genes encoding glycosyltransferases responsible for glycan core extension (Fig. 7, Additional file 1). Infection of PIEs with G9P[13] and G5P[7] significantly affected the expression of genes encoding glycosyltransferases transferring galactose (4 were up- and 3 were downregulated). In contrast to another Golgi-resident *N*-acetylglucosamine transferase family (B3GNT) member—B3GNT6 whose gene expression was downregulated following G9P[13] and RVC infection (Fig. 6), expression of *B3GNT2, B3GNT3* and *B3GNT8*- was significantly upregulated. However, expression of all sialyltransferase-encoding genes (Fig. 7) was mostly downregulated by RVC and both RVA viruses with the exception of *ST3Gal4* and the major Sialyl-Tn synthase—*ST6GalNAc1* whose expression was marginally upregulated after infection with both RVA strains.

**Fig. 8:**
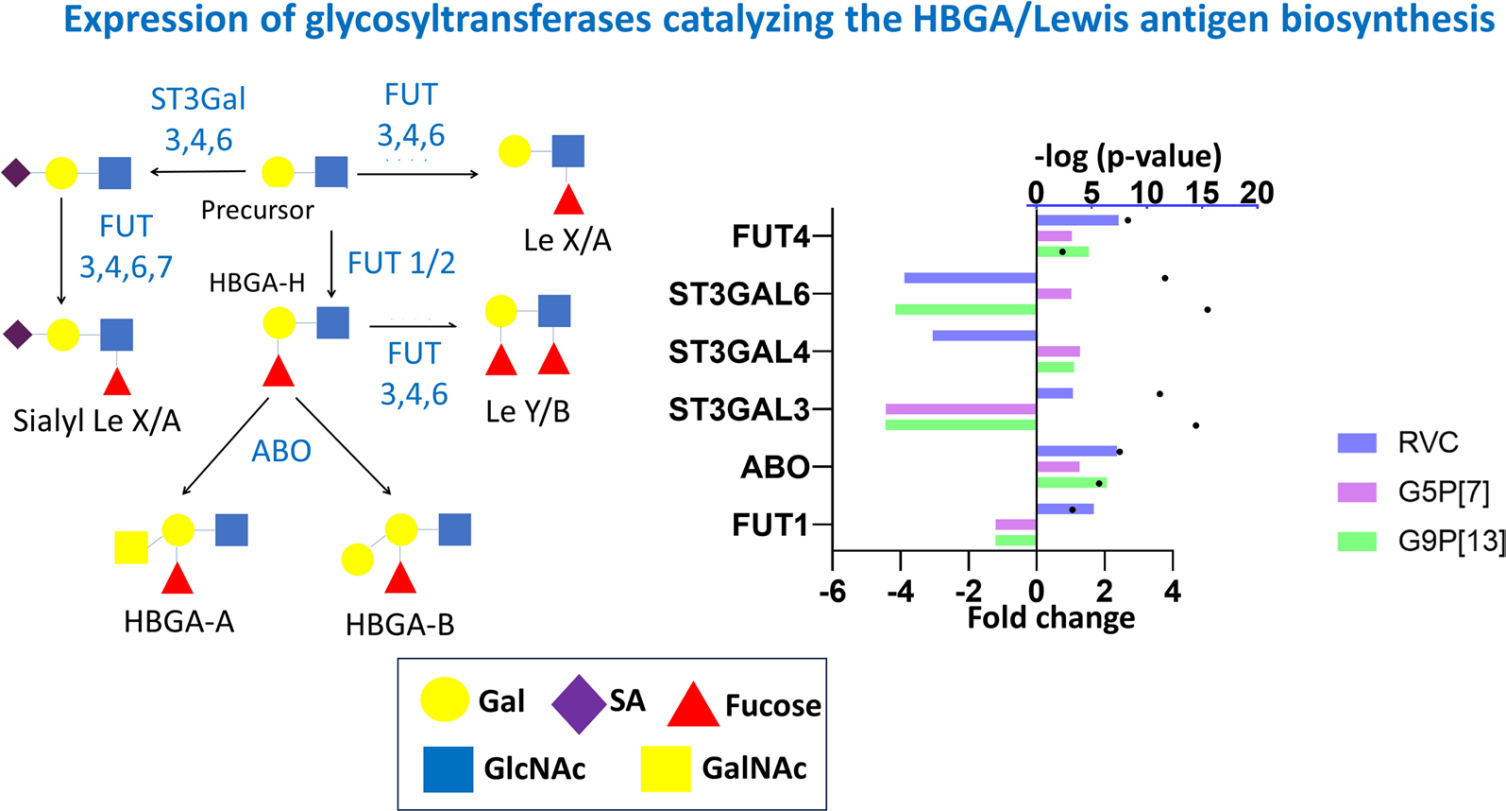
Change of expression of genes encoding glycosyltransferases catalyzing the HBGA/Lewis antigen biosynthesis in PIEs infected with RVC, G9P[13] and G5P[7] versus control (non-infected PIEs). Biosynthesis of all HBGA/Lewis antigens such as A, B, H, Lewis a (Le^a^), Lewis b (Le^b^), Lewis x (Le^x^) and Lewis y (Le^y^) FUT2 is provided by transferring fucose (Fuc), d-galactose (Gal), *N*-acetylgalactosamine (GalNAc), *N*-acetylglucosamine (GlcNAc) and SA residues which is provided by a set of glycosyltransferases. Bars indicate fold change difference between infected PIEs vs uninfected control. Black circles represent the significance (*p*-value < 0.05, i.e. 1.3 in − log10 format)

Analysis of DEGs encoding glycosyltransferases associated with HBGA/Lewis biosynthesis (Fig. 8, Additional file 1) indicated that the expression of fucosyltransferase-encoding gene (*FUT1*) responsible for transfer of fucose to GalNAc was marginally downregulated after G9P[13] and G5P[7] while the infection with RVC led to its significant upregulation. Expression of other genes encoding glycosyltransferases associated with Lewis X/A/Y/B was (ABO, FUT4) was significantly (and RVC) or marginally (G5P[7]) upregulated.

**Fig. 9:**
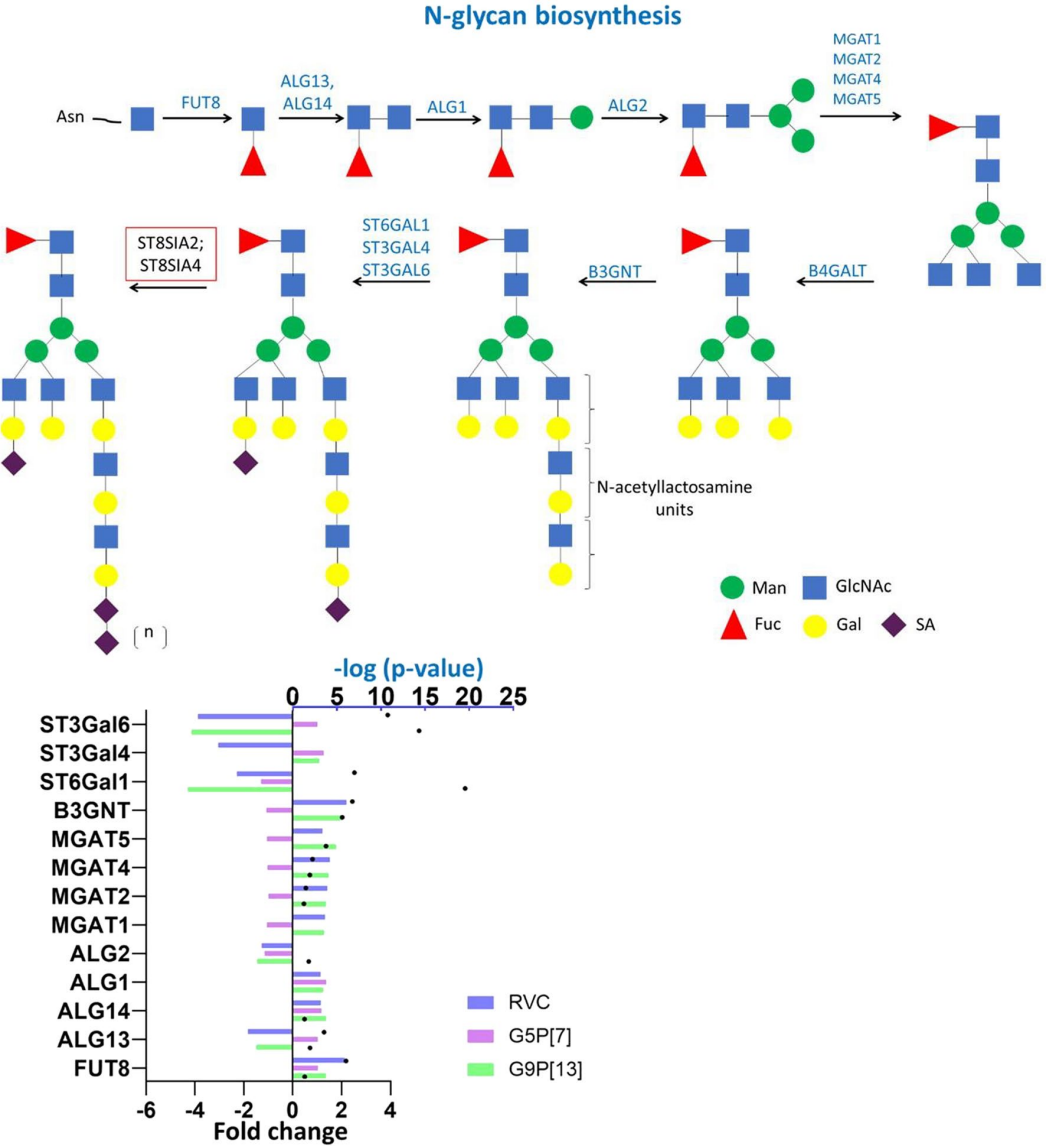
Change of expression of genes encoding glycosyltransferases catalyzing the *N*-glycan biosynthesis in PIEs infected with RVC, G9P[13] and G5P[7] versus control (non-infected PIEs). Similar to *O*-glycans, the *N*-glycan biosynthesis requires presence of multiple glycosyltransferases sometimes with overlapping activity. Bars indicate fold change difference between infected PIEs vs uninfected control. Black circles represent the significance (*p*-value < 0.05, i.e. 1.3 in -log10 format). Red boxes represent genes whose expression was not detected

The expression of most glycosyltransferase (including those transferring mannose, *N*-acetylglucosamine, fucose and galactose)-encoding genes associated with *N*-glycan biosynthesis was significantly upregulated in PIEs infected with G9P[13] and RVC (Fig. 9, Additional file 1). We observed an opposite effect on the expression of genes (*ALG13-*downregulated and *ALG14-*upregulated) encoding two subunits of UDP-*N*-acetylglucosamine transferase following G9P[13] and RVC infection. This data indicates that infection with both RVA and RVC viruses was associated with predicted downregulation of catalytic function of this enzyme while its ability to be recruited to the ER membrane was upregulated [46]. Further extension of this structure is predicted to be inhibited by reduced expression of ALG2-encoding gene (significant effect for G9P[13] and marginal for RVC and G5P[7]). While the next step—addition of *N*-acetylglucosamine to mannose by members of MGAT family was predicted to be activated by RVC and G9P[13] infection, G5P[7] infection led to marginal decrease of MGAT-encoding genes expression. As was mentioned before, we observed the opposite effect of infection on the expression of B4GALT-family glycosyltransferases. The most prominent effect on the modulation of glycosyltransferase-encoding gene expression was upregulation of sialyltransferase-encoding genes whereby infection with G9P[13] and RVC, but not G5P[7] resulted in significant downregulation of these genes.

**Fig. 10:**
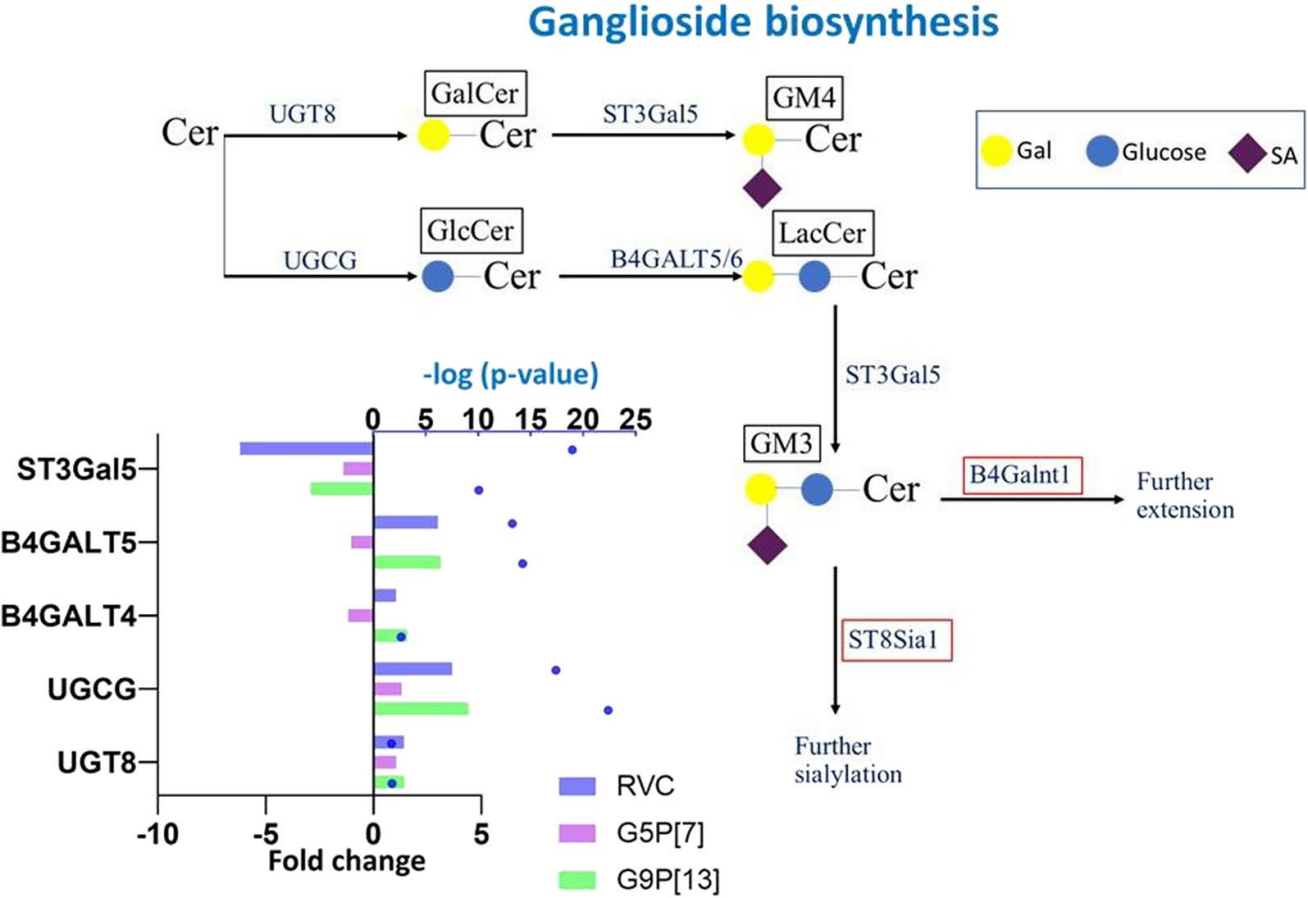
Modulation of expression of genes encoding glycosyltransferases catalyzing the ganglioside biosynthesis in PIEs infected with RVC, G9P[13] and G5P[7] versus control (non-infected PIEs). Bars indicate fold change difference between infected PIEs versus uninfected control. Blue circles represent the significance (*p*-value < 0.05, i.e. 1.3 in − log10 format). Red boxes represent genes whose expression was not detected

All glycosyltransferase (with the exception of sialyltransferase) -encoding genes involved in ceramide biosynthesis (Fig. 10, Additional file 1) were significantly upregulated following infection with the two sialidase-dependent strains (G9P[13] and RVC).

The unique downregulating effect of G9P[13] and RVC infection on the expression of sialyltransferase-encoding genes led us to further analyze the expression of genes associated with SA biosynthesis/degradation (Table 1, Additional file 1). Similar to the modulation of sialyltransferase-encoding genes, infection with G9P[13] and RVC downregulated the expression of SA biosynthesis-encoding genes. More specifically, both aforementioned viruses downregulated the expression of *NANP* gene encoding *N*-acylneuraminate 9-phosphatase responsible for dephosphorylation of sialic acid 9-phosphate to free sialic acid [47]. RVC infection also led to downregulation of expression of gene encoding *N*-acetylneuraminic acid 9-phosphate synthase (Neu5Ac-9-P synthase) which catalyses the primary synthesis of the most common sialic acid, *N*-acetylneuraminic acid (Neu5Ac, NeuNAc, or NeuAc) and *SLC35A1* – gene encoding SA transporter—a transmembrane protein moving SA produced in nucleus into the Golgi apparatus, where SA is used as a substrate for sialylation by sialyltransferases [48, 49]. While no significant effect on this gene expression was observed in PIEs infected with G9P[13], infection with this virus led to downregulation of expression of NAGK-encoding gene whose product provides the conversion of GlcNAc to GlcNAc-6-phosphate [50]. In addition, G9P[13] and RVC infection significantly upregulated the expression of genes encoding proteins associated with SA catabolism with the most prominent change in expression of the gene encoding RENBP enzyme known for its catabolic role in SA metabolism [51]. Similar effect of these viruses was observed for the expression of gene encoding NEU1—sialidase which removes terminal SA from glycans [52]. In addition, G9P[13] infection was associated with downregulation of another catalytic enzyme—NPL. In contrast, no prominent change of genes encoding enzymes responsible for SA biosynthesis/degradation was observed in PIEs infected with G5P[7].

**Table 1:** Modulation of expression of genes associated with SA metabolism in PIEs infected with RVC, G9P[13] and G5P[7]

Gene	RVC		G9P[13]		G5P7	
	Fold change	− log *p* value	Fold change	− log *p* value	Fold change	− log *p* value
*Biosynthesis/activation/transport*						
GNE	− 1.2	ns	− 1.1	ns	− 1.2	ns
NANS	* − 1.4*	**1.9**	1.3	ns	1.1	ns
NANP	* − 2.4*	**6.9**	* − 1.6*	**2.7**	− 1.1	ns
NAGK	1.1	ns	1.5	**2.1**	1.0	ns
CMAS	− 1.2	ns	− 1.0	ns	1.2	ns
SLC35A1	* − 1.9*	**4.5**	− 1.1	ns	− 1.0	ns
*Catabolism/degradation*						
NEU3	1.2	ns	− 1.3	ns	− 1.1	ns
NEU1	2.8	**11.6**	2.3	7.8	− 1.0	ns
SLC17A5	− 1.1	ns	1.1	ns	1.1	ns
SIAE	1.1	ns	1.3	ns	1.0	ns
NPL	− 1.1	ns	* − 1.5*	1.7	− 1.0	ns
CTSA	1.18	ns	1.2	ns	− 1.1	ns
RENBP	4.6	**18.5**	5.5	23.8	− 1.1	ns

Colors are indicated where Italic is significantly downregulated, and underline is significantly upregulated. Bold font indicates statistical significance

## Discussion

Our study indicated the overall more prominent alteration of gene expression in PIEs infected with RVC strain and RVA G9P[13] compared to infection with G5P[7]. To compare the innate immune signaling in PIEs to RVC and RVA infection we analyzed the expression of innate immune response related DEGs. Although, the role of IFNs especially type I and III in protection against RVAs has been recognized previously, while no previous study has been done to investigate IFN response following RVC infection [53, 54]. Canonical pathway and DEG analyses conducted in this study demonstrated a robust upregulation of IFN response in PIEs infected with RVC vs RVA [55, 56]. In addition, we found a principal difference in modulation of RIG1 – a key molecule that recognizes virus-infected cells and promotes the activation of the antiviral program [57]. Significant downregulation of RIG1-encoding gene in G5P[7] infected PIEs supports the previous evidences where NSP1 of a RVA strain was shown to inhibit type I IFN responses through downregulation of RIG-I expression [58]]. The unique profile of DEGs associated with innate immune response following RVC infection was not limited to virus-recognizing molecules but also involved significant upregulation of interferon-stimulated genes *MX-1* and *OAS1*. Thus, the robust upregulation of the genes encoding components of the innate immune system in PIEs infected with RVC may provide an explanation for less efficient replication of RVC in vitro compared to G5P[7]. However, more research is needed to test the hypothesis that the robust immune response plays a role in poor replication of RVC in cell culture. Besides, RVC infection has been shown to affect neonates indicating an unique age-specific characteristic of RVC infection [5–7]. In general, the age specificity of RVs is thought to be unrelated to receptor expression indicating that other host factors can contribute to this effect [59]. Studies have shown that the overall lower activity of the innate immune system in neonates [60]. Thus, while in older animals RVC can be efficiently recognized by the components of the innate immunity, relatively low activity of this system (including RIG-1) may facilitate RVC replication. An increased susceptibility of young children to severe respiratory-syncytial virus (RSV) disease was shown to be associated with the attenuated RIG-I-dependent IFN-α responses [61].

Intestinal mucins have been considered as a key components of the innate immune system [62, 63]. In our study we comparatively evaluated the effect of RVA or RVC infection on expression of genes encoding transmembrane and secreted mucins and genes encoding different families of glucosyltransferases [64]—responsible for glycan-core development [65] and extension resulting in development of a variety of ABO blood group and Lewis antigens [66–69]. It is broadly accepted that virus infection of epithelial cells is generally associated with upregulation of transmembrane mucin-encoding genes suggesting their protective role [70, 71] which was previously demonstrated to generate an anti-inflammatory state following infection with the respiratory syncytial virus [72]. Thus, while the upregulation of the transmembrane mucin-encoding genes observed for RVA (G9P[13]) and RVC may be considered as a mechanism of cellular protection, overall marginal downregulation (significant for MUC12 only) of these genes by RVA (G5P[7]) suggests a unique inhibitory strain-specific effect of this virus allowing it to penetrate the mucus layer more efficiently.

Expression profile of genes encoding another group of mucins—secretory—was also impaired but to a lesser extent. RVA infection in mice is associated with decreased levels of MUC2-positive goblet cells [29]. However, the same study did not identify changes in MUC2 mRNA levels after infection with a simian RVA strain. While the protective role of the transmembrane mucins is strongly associated with signaling pathways, secreted mucins are not linked to cellular membrane and their major role in protection against pathogens is provided by the ability to form a large, net-like polymer structures and by *O*-glycans serving as decoy epitopes for pathogen binding [73]. Significant upregulation of the genes encoding for secreted mucins in PIEs infected with RVC and RVA (G9P[13]) but not G5P[7] is suggestive of the possible strain-specific effects In addition, with the overall similarity of the modulation of core 2 synthases of gel-forming mucins in RV infection.

The protective role of mucins against RV infection has been linked to the mucin-associated carbohydrates – glycans, however, the data on the role of RV infection on mucin glycosylation profile is limited [63]. For example, one study demonstrated a stimulatory effect of RVA on intestinal mucin glycosylation [31]. Interestingly, our data demonstrated the ability of RV (RVC and G9P[13]) to downregulate the expression of gene encoding B3GNT6 thus potentially affecting the abundance of major *O*-glycans found on MUC2 in intestine [74]. In contrast, expression of glycan cores 1, 2 was predicted to be activated after infection with these RVs. Previously RVAs of different origin have been shown to possess the specific recognition of glycan cores 2,4 and 6 [75, 76].

Interestingly, similar to RVA (G9P[13]) [24, 25], RVC infection is associated with a predicted downregulation of SA biosynthesis/activation/transfer while SA degradation was predicted to be upregulated. Thus, infection with these two sialidase-sensitive RVA and RVC viruses tended to decrease the overall metabolism of SA. Glycoproteins and glycolipids often terminate in SAs, protecting them from interaction with enzymatic and non-enzymatic [77] proteins(lectins). Thus, our data suggest that predicted reduction of SA expression increases the availability of non-sialylated glycans for RV attachment [23]. Besides, the function of SA is not limited to its direct interactions with RV particles. Absence of SA affects the function of the host cell–cell interaction, signaling, carbohydrate-protein interactions, cellular aggregation, immune response and may be associated with cancer development [78, 79].

Analysis of the expression of genes encoding glycosyltransferases responsible for transfer of other sugars (galactose, fucose, mannose, *N*-acetyl glucosamine and *N*-acetyl galactosamine) associated with *O*-, *N*-glycan and glycolipid biosynthesis indicated a similar pattern of modulation by RVC and G9P[13] suggesting a common pathway affected in IECs following infection with sialidase-sensitive RVA and RVC viruses. Our findings indicate that modulation of the intestinal glycosylation profile may be an alternative strategy to control RVA and RVC infection.

### Supplementary Information

**Additional file 1. **Differential gene expression data.

## Data Availability

The datasets supporting the conclusions of this article are included within the article and its additional files.
